# DEHP induces stress urinary incontinence by causing urethral sphincter dysfunction via inflammation and apoptosis: a mechanism study based on animal experiments, network toxicology, and molecular docking

**DOI:** 10.3389/fcell.2026.1866437

**Published:** 2026-06-22

**Authors:** Xiaozhi Xia, Tao Liu, Wei Wang, Yin Tang, Tao Jin

**Affiliations:** 1 Department of Urology, Institute of Urology, West China Hospital, Sichuan University, Chengdu, Sichuan, China; 2 Department of Urology, West China Tianfu Hospital, Sichuan University, Chengdu, Sichuan, China

**Keywords:** DEHP, molecular docking, network toxicology, SUI, urology

## Abstract

**Objective:**

Di (2-ethylhexyl) phthalate (DEHP), a pervasive environmental pollutant, poses significant health risks, yet its role in stress urinary incontinence (SUI) remains largely unexplored.

**Methods:**

This study integrated network toxicology, molecular docking, and animal experiments to investigate DEHP-induced SUI mechanisms.

**Results:**

Through cross-analysis of multiple public databases, we identified 95 potential targets linking DEHP exposure to SUI. GO and KEGG enrichment analyses revealed that these targets are primarily involved in immune-related pathways, including apoptosis, TNF signaling, NOD-like receptor signaling, and inflammatory mediator regulation of TRP channels. Using protein interaction network analysis, we further identified five core targets (NFKB1, CASP3, BCL2, TLR4, MMP9). Molecular docking confirmed strong binding affinities between DEHP and these proteins. In vivo experiments demonstrated that DEHP exposure induces urethral sphincter damage and degeneration in rats, thereby accelerating SUI progression.

**Conclusion:**

This study elucidates the molecular mechanisms underlying DEHP-induced SUI and highlights network toxicology as a powerful approach for assessing health risks of environmental contaminants.

## 1 Introduction

Di (2-ethylhexyl) phthalate (DEHP), a key phthalate ester and commonly used plasticizer in polyvinyl chloride (PVC) products (Erythropel et al., 2014), poses a significant environmental and health risk. Due to its non-covalent bonding to plastics, DEHP can easily leach into the surroundings during production, use, and disposal, leading to human exposure through ingestion, inhalation, and skin contact (Yang et al., 2022; Wu et al., 2021). Accumulating evidence links DEHP to various health issues, including cardiovascular (Wen et al., 2022) and reproductive diseases (Li et al., 2024), as well as tumors (Li et al., 2025). Moreover, it has been associated with urinary system disorders, such as hypospadias (Zhu et al., 2023), bladder cancer (Lin et al., 2025), prostatic hyperplasia (Yang et al., 2022), and prostate cancer (Yin et al., 2025), highlighting its broad health impacts.

Stress urinary incontinence (SUI) is the predominant form of urinary incontinence, characterized by unintentional urine leakage caused by increased intra-abdominal pressure during activities such as physical exertion, exercise, coughing, or sneezing (D'Ancona et al., 2019). Research focusing on the elderly reveals that over 20% suffer from urinary incontinence, significantly impairing their physical and mental wellbeing (Ko et al., 2005). The onset of SUI is multifactorial, involving neurogenic (Yoshimura and Miyazato, 2012), myogenic (Koch and Kaufman, 2022), connective tissue (Ulmsten and Falconer, 1999), and hormonal factors (Castelán et al., 2020), among others. Notably, the activation of inflammatory pathways play pivotal roles in its development (Post et al., 2022). In a rat model of SUI induced by mechanical injury, fibroblasts (Liu et al., 2018) exhibit heightened apoptosis and oxidative damage, further underscoring these mechanisms.

DEHP metabolites are frequently found in human urine (Wang et al., 2022), indicating widespread exposure. Research reveals that DEHP harms the body by generating reactive oxygen species (Huang et al., 2019), inducing pro-inflammatory cytokines (Jepsen et al., 2004), and disrupting hormone functions (Yin et al., 2025)—factors also involved in the development of SUI (Post et al., 2022). Despite mounting evidence linking DEHP to health issues, the exact mechanisms behind its role in SUI’s onset and progression remain unclear.

Considering DEHP’s pervasive presence in daily life and its potential health risks, understanding its role in SUI is crucial. We employed a multifaceted approach, integrating computational predictions with experimental validation. Initially, network toxicology and molecular docking techniques were used to identify potential molecular targets and pathways involved in DEHP-induced SUI. Subsequently, an SUI rat model was established to evaluate the direct impact of DEHP exposure on urethral sphincter function.

## 2 Methods

### 2.1 Animal and grouping

We created a SUI animal model in 6–8 week-old male SPF Sprague-Dawley rats by electrocauterizing bilateral urethral sphincters (Khodari et al., 2012; Chermansky et al., 2004). Rats were anesthetized via isoflurane inhalation and placed supine with secured limbs. After shaving the lower abdominal hair and disinfecting the area with iodophor, a midline incision was made. Tissues were bluntly separated to access the bladder and prostate. The prostate was then separated to reveal the striated urethral sphincter. Each side of the sphincter underwent 1-s electrocautery, and the incision was closed with 3–0 absorbable sutures.

The rats were randomly split into three groups, control (corn oil, 1 ml/kg, 21-day gavage), model (electrocautery + corn oil, same dosage and duration), and model + DEHP (electrocautery + DEHP 1000 mg/kg/day in corn oil, 21-day oral administration), with 8 rats per group. Rats in the control group underwent sham surgery, which involved only abdominal incision and exposure of the urethral sphincter. After surgery, enrofloxacin (5 mg/kg/day) was given subcutaneously for 3 days to prevent infection. The rats were kept at 21° C–22  C, 40%–60% humidity, under a 12-h light/dark cycle, with unrestricted food and water.

### 2.2 Identification of potential toxicity targets for DEHP

The SMILES and molecular structure of DEHP were retrieved from PubChem by searching “Di (2-ethylhexyl) phthalate.” Using this data, potential toxicity targets were identified via SwissTargetPrediction and SEA search server. After cross-referencing with Uniprot, non-human genes, invalid duplicates were removed, and standardized gene names were obtained.

### 2.3 Obtain targets related to SUI

Relevant disease targets for SUI were retrieved from GeneCard and OMIM databases by entering the keyword. These targets were then merged and duplicates removed. Overlay drug component targets with disease targets, identify intersecting genes via Venn diagram.

### 2.4 Target protein interaction network construction

To delve deeper into DEHP-induced protein-protein interactions linked to diseases, drug-intersection genes were submitted to the String database (https,//string-db.org/) to construct a protein-protein interaction (PPI) network (Nithya et al., 2026). The species was set to “*Homo sapiens*,” and the minimum interaction score was adjusted to 0.4 for reliability, with other parameters unchanged. Results were saved in TSV format and imported into Cytoscape 3.8.2 for network analysis. The analysis results were saved, and node size and color were adjusted to reflect Degree values, with larger nodes indicating higher Degree values. This process yielded a comprehensive protein-protein interaction network map.

### 2.5 Go enrichment analysis and KEGG pathway analysis

Upload drug-disease intersection genes to DAVID (https,//david.ncifcrf.gov/summary.jsp), choosing OFFICIAL_GENE_SYMBOL for gene identifiers and *Homo Sapiens* as the species. Utilize DAVID 6.8 GO to annotate target proteins’ roles in DEHP-induced diseases across Biological Process (BP), Cellular Component (CC), and Molecular Function (MF). Conduct KEGG pathway enrichment analysis to identify disease-related signaling pathways. We selected the top 10 GO functions in BP, CC, and MF, along with 20 relevant KEGG pathways sorted by P-value indescending order, as key enrichment processes and signaling pathways, to predict DEHP’s disease-causing mechanisms.

### 2.6 Molecular docking of DEHP with key target proteins

In network analysis, proteins with higher Degree values typically hold more significant positions, playing crucial roles in DEHP-induced SUI. To verify the interaction between DEHP and key targets, molecular docking (Li et al., 2022; Wang et al., 2025; Zhang et al., 2025) was performed using AutoDock Vina (1.1.2). The steps are as follows, First, download DEHP compounds in SDF format from PubChem, optimize their energy in ChemBio3D,and then prepare them for docking in AutoDockTools-1.5.6 by adding hydrogens, calculating charges, and setting rotatable bonds, saving as “pdbqt” files. Next, download key target proteins from the PDB database, prioritizing human proteins with high resolution and ligands structurally similar to DEHP. Import these proteins into PyMOL to remove original ligands and water molecules, then further process them in AutoDockTools for hydrogen addition, charge calculation, atom type specification, and saving as “pdbqt” files. For docking, use the original ligand’s position as the box center, if absent, use key amino acid residues’ vicinity. Set the grid box to 60 × 60 × 60 with 0.375 Å spacing, keeping other parameters default. Finally, analyze interactions using PyMOL 2.3.0 and Ligplot V2.2.8.

### 2.7 Determination of leak point pressure (LPP) and histological assay of rats

We assessed the condition of SUI in DEHP-fed rats by measuring LPP, and the method can be found in the following literature (Jaskowak and Danziger, 2023) LPP refers to the intravesical pressure at which urine leakage begins。The lower the LPP value,the more severe the incontinence tends to be.

After measuring LPP, we euthanized the rats and collected their urethral sphincter specimens for histological examination. We assessed the injury of urinary sphincter muscle through Immunofluorescence and muscle fibrosis through Sirius Red staining. The antibody used was the Fast skeletal heavy chain antibody (Affinity, catalog number:DF13212).

## 3 Results

### 3.1 Hub genes prediction

Using a Probability >0 threshold in SwissTargetPrediction,113 targets were identified. Additionally, searching for DEHP in the SEA server yielded 139 targets. After removing duplicates, a total of 246 component targets were obtained.

GeneCards and OMIM databases provided 4,452 and 55 SUI targets, respectively. Combining these and removing duplicates yielded a total of 4,497 unique genes.

**FIGURE 1:**
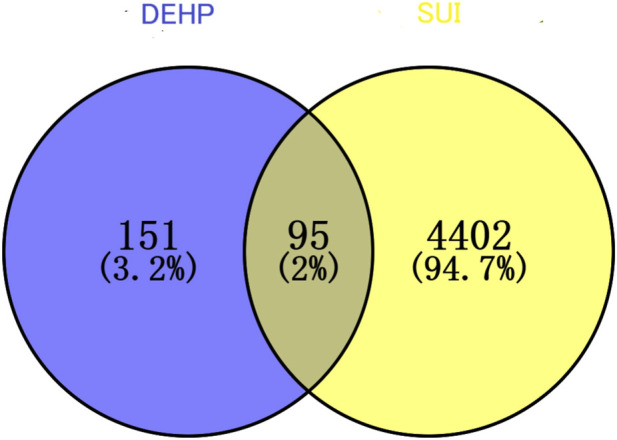
The Venn diagram illustrating the targets of DEHP and SUI.

By intersecting DEHP target genes with SUI-related genes,95 overlapping target genes were identified. These hub genes represent the potential targets through which DEHP exposure may induce SUI (Figure 1).

### 3.2 Interaction network of potential targets and acquisition of core genes

**FIGURE 2:**
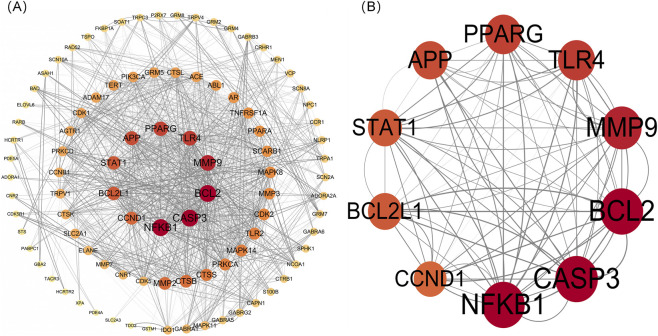
PPI network analysis diagram **(A)** The PPI network of potential targets for DEHP exposure caused SUI. **(B)** The Top 10 Hub Genes Identified from the PPI Network. The redder the node, the greater its degree value.

To delve deeper into the mechanisms and key targets of DEHP-induced SUI, a protein-protein interaction (PPI) network was built using the intersecting targets. With a confidence threshold of 0.4,the network included 95 nodes and 1,262 edges, revealing intricate interactions among all targets (Supplementary Table S1). These interactions suggest their involvement in DEHP-related urethral sphincter damage (Figure 2A). The top ten core targets identified were NFKB1, CASP3, BCL2, MMP9, TLR4, PPARG, APP, STAT1, BCL2L1 and CCND1 (Figure 2B).

### 3.3 Target function analysis and pathway enrichment analysis

**FIGURE 3:**
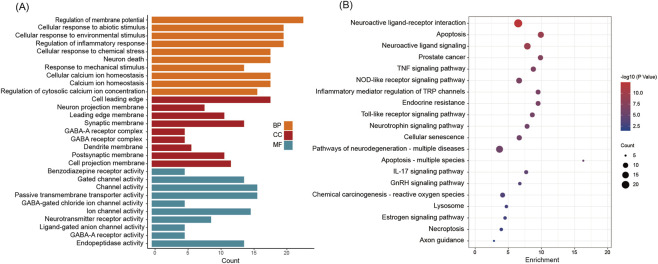
Target function analysis and pathway enrichment analysis **(A)** Top 10 GO terms in each of the three GO categories (BP, CC, and MF) from the enrichment analysis of the target genes. **(B)** Top 20 pathways of the target genes in the KEGG enrichment analysis. In the bubble plot, the x-axis represents the number of genes enriched in each pathway. The size of the bubbles corresponds to the count of genes enriched in the respective pathways, while the color intensity reflects the statistical significance of the enrichment.

To uncover the functions and pathways involved in DEHP-induced SUI, a functional analysis of potential targets was conducted (Figure 3; Supplementary Tables S2-S4). The top 10 GO pathways are shown in Figure 3A. Notably, signal transduction, pathways related to ion homeostasis (especially cellular calcium ion homeostasis), cell membrane injury, cellular responses to chemical stress, and inflammatory responses may play crucial roles in SUI caused by DEHP exposure.

The enrichment pathways through which DEHP induces SUI were identified, involving 224 pathways (Supplementary Table S5). The top 20 KEGG pathways, including apoptosis, TNF signaling, NOD-like receptor signaling, and inflammatory mediator regulation of TRP channels, are shown in Figure 3B. These findings suggest DEHP exposure may lead to urinary sphincter dysfunction via apoptosis, inflammation, and chemokine signaling.

### 3.4 Molecular docking verification

Based on prior analysis, the top five critical targets were chosen for semi-flexible docking with DEHP. The binding affinity measures how effectively DEHP binds to the target protein. A binding affinity below zero indicates free binding, with lower values suggesting a higher likelihood of interaction.

**FIGURE 4:**
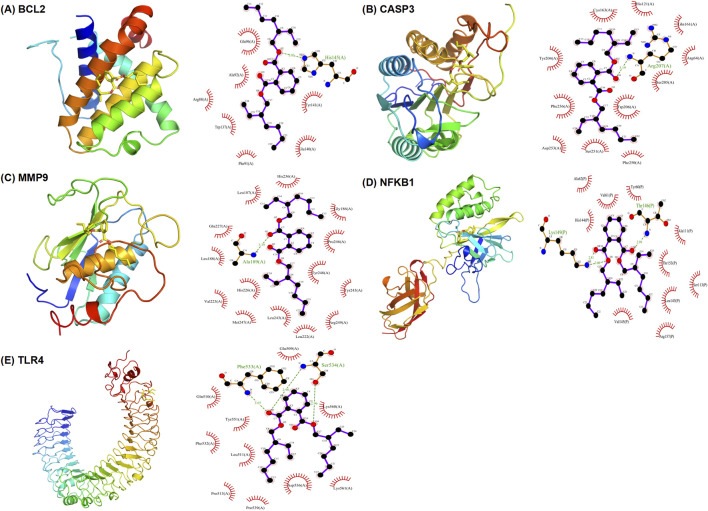
Molecular docking. DEHP with **(A)** BCL2 **(B)** CASP3 **(C)** MMP9 **(D)** NFKB1 **(E)** TLR4 binding.

Docking analysis reveals that DEHP effectively binds to the active sites of target proteins, as depicted in Figure 4 and detailed in Supplementary Table S6. DEHP forms a hydrogen bond with His145 in BCL2 at a 3.03 Å bond length, with Arg207 in CASP3 at 3.19 Å, and with Ala189 in MMP9 at 3.39 Å. In NFKB1, it binds to Lys149 and Thr146 with bond lengths of 2.81 Å,3.00 Å, and 2.99 Å, respectively. For TLR4, DEHP forms hydrogen bonds with Ser534 and Phe533 at 2.96 Å and 3.07 Å. Additionally, DEHP establishes strong hydrophobic interactions with nearby amino acid residues. In conclusion, DEHP shows high affinity for these molecules. Comprehensive docking results are provided in Table 1.

**TABLE 1:** Docking results of DEHP with core target proteins.

Target proteins	PDB ID	Compound	Binding energy (kcal/mol)
BCL2	5FCG	DEHP	−5.9
CASP3	4JJE		−6.1
MMP9	4XCT		−8.1
NFKB1	1SVC		−5.3
TLR4	4G8A		−6.1

### 3.5 The effects of DEHP on the urinary sphincter of Sprague-dawley rats

**FIGURE 5:**
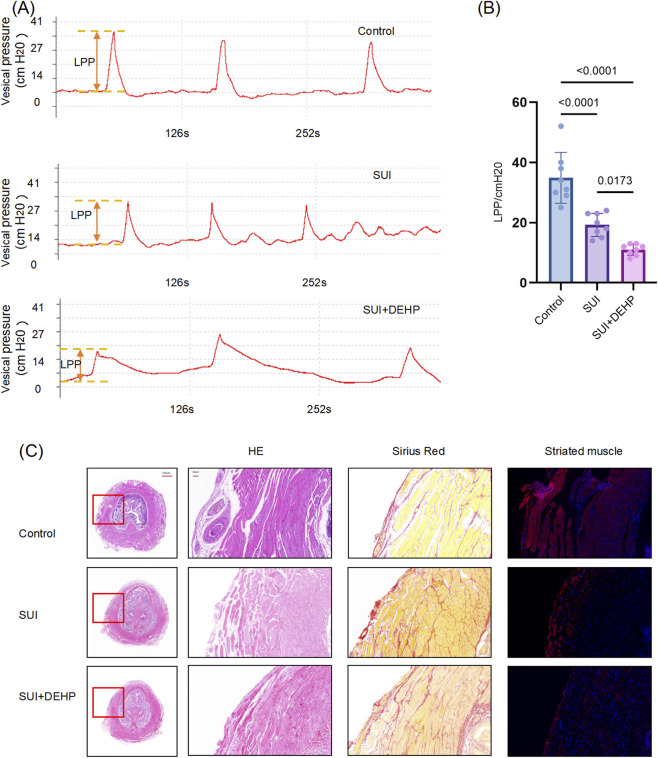
LPP test and histological staining of urethral sphincter tissue to assess the exacerbated damage caused by DEHP on electrically cauterized urethral sphincter. **(A,B)** LPP test showed that DEHP worsened stress urinary tract symptoms in rats. **(C)** HE and Sirius Red staining showed that DEHP exacerbated destruction and fibrosis of the urethral sphincter muscle striated muscle:Blue: DAPI Red: Fast Myosin Skeletal Heavy chain A.ntibody.

The LPP values in the bladders of DEHP-exposed rats were significantly lower than those in the model and control groups (Figure 5AB), indicating that DEHP worsened lower urinary tract symptoms. Tissue staining and immunofluorescence revealed severe urinary sphincter muscle fibrosis and damage in the DEHP group compared to the others (Figure 5C). These evidences suggest that DEHP exacerbates SUI by damaging the urethral sphincter muscle and causes fibrosis.

## 4 Discussion

Environmental factors are increasingly recognized as significant contributors to health issues (Sundas et al., 2024),with plasticizers like DEHP being widely used (Nagorka et al., 2022) and leading to inevitable long-term exposure. Humans are primarily exposed to DEHP through dietary intake, particularly from packaged foods and beverages. Epidemiological studies (Gaudin et al., 2008) show that urine samples from exposed workers had median DEHP metabolite concentrations of 16.1 μg/L before shifts and 55.9 μg/L after, This study utilized network toxicology, integrating data from SwissTargetPrediction, OMIM, and GeneCards, to identify 95 potential targets linking DEHP to SUI.PPI network analysis pinpointed five core targets—NFKB1,CASP3,BCL2,MMP9,and TLR4—as central mediators. KEGG and GO analyses revealed key mechanisms, while molecular docking confirmed strong binding affinities between DEHP and these targets, supporting their role in DEHP-induced SUI. Our rat experiments demonstrated that DEHP intake worsens urinary incontinence symptoms, suggesting it may trigger SUI by causing inflammation and urethral sphincter damage.

The transcription factor NF-κB is crucial for regulating innate and adaptive immune functions and mediating inflammatory responses (Liu et al., 2017),while Toll-like receptors (TLRs) are key pattern recognition receptors that initiate innate immune responses (Duan et al., 2022). Matrix metalloproteinase 9 (MMP9) also plays a significant role in inflammation and cell migration (Fang et al., 2025). Research has highlighted the inflammatory role of the TLR4-NFKB1 pathway in neurodegenerative diseases (Sharma et al., 2024). Our enrichment analysis further underscores the involvement of inflammatory and immune pathways, such as TNF signaling, NOD-like receptor signaling, and inflammatory mediator regulation of TRP channels. We observed destruction of the urethral sphincter tissues of DEHP-fed rats, suggesting that DEHP may induce immune responses, triggering muscle inflammation and leading to urinary sphincter damage and SUI by binding to these molecules.

Caspases are key regulators of apoptosis (Porter and Jänicke, 1999), aspase-3 (CASP3) is a primary death protease responsible for cleaving essential cellular proteins. Studies indicate that CASP3 upregulation may be linked to muscle fibrosis (Tanaka et al., 2022). And the BCL2 protein family controls apoptosis by regulating cytochrome c release from mitochondria (Vogler et al., 2025). One research showed that BCL2 upregulation in skeletal muscle cells marks an early myogenic stage, inhibiting apoptosis and promoting clonal expansion (Dominov et al., 1998). Here, DEHP may bind to BCL2 and silence its expression, thereby inducing apoptosis. Our enrichment analysis highlights apoptosis as a critical factor in DEHP-induced SUI, suggesting DEHP triggers cell death pathways that contribute to the condition.

As chemical product exposure rises, toxic substances have become a critical factor in urinary system disease development. Our research offers vital insights into chemical-induced SUI, laying the groundwork for future studies. We found that DEHP damages the urethral sphincter in animals, causing lower urinary tract symptoms. By integrating network pharmacology and molecular docking, we explored DEHP’s mechanism in inducing SUI. While our study established a computational framework prioritizing molecular mechanisms linking DEHP exposure to SUI progression, it also has limitations, such as the need for further experimental validation to confirm the identified pathways and targets.

Our research relies on computational models, while insightful, cannot fully represent the complexities of long-term, low-dose exposure scenarios or real-life human interactions with microplastics. Molecular docking, useful for simulating target interactions under specific conditions, falls short in replicating the dynamic *in vivo* environment, including protein conformation changes, the cellular microenvironment, and competitive binding by endogenous ligands. To address these limitations, systematic experimental validation using physiologically relevant animal models and *in vitro* cell models is essential to fill the gaps in our understanding of these mechanisms.

In conclusion, our study identified BCL2, NFKB1, TLR4, MMP9, and CASP3 as key genes linking DEHP exposure to SUI, likely involved in inflammatory and apoptotic pathways. This was supported by observed fibrosis and destructionof urinary sphincter muscle in the urethral sphincter of DEHP-exposed rats.

## Data Availability

The original contributions presented in the study are included in the article/Supplementary Material, further inquiries can be directed to the corresponding authors.
